# Comparison of the diagnostic performance of NBI, Laser-BLI and LED-BLI: a randomized controlled noninferiority trial

**DOI:** 10.1007/s00464-022-09197-8

**Published:** 2022-04-11

**Authors:** Takuma Higurashi, Keiichi Ashikari, Shigeki Tamura, Tomohiro Takatsu, Noboru Misawa, Tsutomu Yoshihara, Yuki Ninomiya, Yuki Okamoto, Masataka Taguri, Taku Sakamoto, Shiro Oka, Atsushi Nakajima, Shinji Tanaka, Takahisa Matsuda

**Affiliations:** 1 Department of Gastroenterology and Hepatology, Yokohama City University School of Medicine, 3-9 Fukuura, Kanazawa-ku, Yokohama, 236-0004 Japan; 2 Department of Endoscopy, Hiroshima University Hospital, Hiroshima, Japan; 3 Department of Data Science, Yokohama City University School of Data Science, Yokohama, Japan; 4 Endoscopy division, National Cancer Center Hospital, Tokyo, Japan

**Keywords:** NBI; Laser-BLI, LED-BLI, Colon tumor, Diagnosis accuracy, NICE classification, JNET classification

## Abstract

**Background and aims:**

New image-enhanced endoscopy (IEE), blue Light Imaging (LED-BLI) is launched in USA and Europe, whereas Blue Laser Imaging (Laser-BLI) is available only Asian and some countries. No studies have directly compared the diagnostic accuracy of narrow band imaging (NBI), Laser-BLI and LED-BLI for colorectal tumors. The present study aimed to compare the diagnostic accuracy of the three methods for colorectal tumor using the NBI international colorectal endoscopic (NICE) classification and the Japanese NBI Expert Team (JNET) classifications.

**Methods:**

This was a multi-center evaluator-blinded, randomized control trial of patients who underwent endoscopic colorectal tumor resection. The patients were randomly assigned to NBI, Laser-BLI or LED-BLI. Cropped images were sent to blinded external evaluators and diagnosed according to NICE and JNET classifications. The diagnostic accuracy of each endoscopy system was compared with non-inferiority test.

**Results:**

A total of 619 colonic tumors were resected from 230 patients and evaluated by external four evaluators. The diagnostic accuracy of NBI for NICE 1, NICE 2, NICE 3 was 90.6%, 90.3% and 99.5%, respectively and for JNET 1, JNET 2A, JNET 2B and JNET 3, it was 94.6%, 72.0%, 79.2% and 99.1%, respectively. In non-inferiority test, Laser-BLI and LED-BLI revealed non-inferiority to NBI in all NICE and JNET categories (*p*<0.001).

**Conclusions:**

Laser-BLI and LED-BLI had high diagnostic accuracy and non-inferiority of NBI, especially for hyperplastic polyp/sessile serrated lesion and low-grade dysplasia. This is first trial to compare the diagnostic accuracy with NBI, Laser-BLI and LED-BLI and useful to understand the position of each IEE. This trial was registered as UMIN000032107.

**Supplementary Information:**

The online version contains supplementary material available at 10.1007/s00464-022-09197-8.

Image-enhanced endoscopy (IEE) is useful for improving the qualitative diagnosis of colorectal tumors, and knowledge of it has increased in recent years. IEE involves various means of enhancing contrast during endoscopy, using equipment, to improve visualization of lesions and potentially gain insight into the pathology of the lesions [1]. So far, the method with the highest diagnostic accuracy is the pit pattern classification using crystal violet, as reported by Kudo et al. [2]. However, crystal violet staining and magnified observation are costly, time-consuming and labor-intensive; therefore, simpler methods are required. Narrow band imaging (NBI), one of the optical digital methods of IEE, was launched in 2005 by Olympus Medical Systems [3]. Since then, in most countries where gastrointestinal endoscopies are performed, NBI is one of the most frequently used optical digital methods of performing IEE. There are many reports on the usefulness of NBI for detection and diagnosis of colorectal neoplasm. The NBI international colorectal endoscopic (NICE) classification has been proposed and has been reported to be effective for colorectal lesions [4, 5]. The NICE classification is divided into Type 1-3, Type 1 indicates non-neoplastic lesions, such as hyperplastic polyp (HP) and sessile serrated lesions (SSL); Type 2 indicates adenomatous lesions; and Type 3 indicates deep submucosal (d-SM) invasive carcinoma (Supplemental Figure 1). However, NICE Type 2 includes from low-grade dysplasia (LGD) to intramucosal carcinoma and shallow submucosal (s-SM) invasive carcinoma, and it is difficult to differentiate high-grade dysplasia (HGD) or s-SM carcinoma from LGD. Clinically, it is important to resect intramucosal carcinoma and s-SM invasive carcinoma *en-bloc* by endoscopic mucosal resection (EMR) or endoscopic submucosal dissection (ESD). This is because these lesions sometimes have vascular or lymphatic invasion or lymph node metastasis and require accurate pathological diagnosis. To resolve this issue, the Japanese NBI Expert Team (JNET) of Japanese magnifying colonoscopists was organized in 2011. Going through repeated detailed discussion and a web-based prospective trial, JNET achieved consensus regarding NBI classification, and a new NBI colorectal magnifying classification (the JNET classification) was proposed in 2014 [6]. The JNET classifies colorectal lesions into four types based on vessel and surface patterns. (Supplemental Figure 2). The NICE and JNET classifications have both improved the quality of endoscopy and many endoscopists have used these methods.

Recently, other IEE methods have been developed such as, Blue Laser Imaging (Laser-BLI) in 2012, and Blue Light Imaging using light emitting diode (LED) (LED-BLI) (both Fujifilm) between 2016 and 2017 in the USA and Europe, where LASER endoscopes have not been approved for use [7]. Several studies reported the efficacy of Laser-BLI and LED-BLI using NICE and JNET classifications [8–10]. However, no studies have directly compared the diagnostic accuracy of NBI, Laser-BLI and LED-BLI. Thus, many endoscopists and physicians find it hard to interpret the results and determine which IEE method is superior, because all studies had different patient backgrounds and tumor characteristics. This led us to conduct the present trial to compare the diagnostic accuracy of NBI, Laser-BLI and LED-BLI for colorectal tumors using NICE and JNET classifications. Each method had previously reported having high diagnostic accuracy, we planned noninferior test that Laser-BLI and LED-BLI against NBI.

## Methods

### Study design and setting

This study is a multi-center evaluator-blinded randomized controlled noninferiority trial in patients who underwent endoscopic resection (ER) of colorectal tumors. Study coordination, registration and data collection were conducted in the Department of Gastroenterology and Hepatology at Yokohama City University (YCU) Hospital.

### Ethical considerations and registration

The study protocol complied with the Declaration of Helsinki [11] and the Ethics Guidelines for Clinical Research published by the Ministry of Health, Labor, and Welfare, Japan [12]. We obtained approval for this study from the Ethics Committee of Yokohama City University Hospital on April 2, 2018 (B170610004). The protocol and informed consent form were approved by the institutional ethics committee at Yokohama City University Hospital. This trial was registered in the University Hospital Medical Information Network (UMIN) Clinical Trials Registry as UMIN000032107. Written informed consent for participation in the study was obtained from all participating patients. The trial results were reported in conformity with the Consolidated Standards of Reporting Trials (CONSORT) 2010 guidelines [13].

### Participants

We recruited all adult patients visiting the hospital between April 2018 and December 2019 for ER of colorectal tumors. The inclusion criteria were as follows: (1) age 20 years or more on the date of informed consent; (2) patients with colorectal tumors who were undergoing ER; (3) willingness to participate in the study.

The exclusion criteria were as follows: (1) colorectal lesions that were not appropriate to categorize by NICE and JNET classifications; (2) patients judged by the investigators as inappropriate candidates for the trial.

Written informed consent for participation in the study was obtained from all the participating patients.

### Endoscopic Systems

#### NBI

**Fig. 1:**
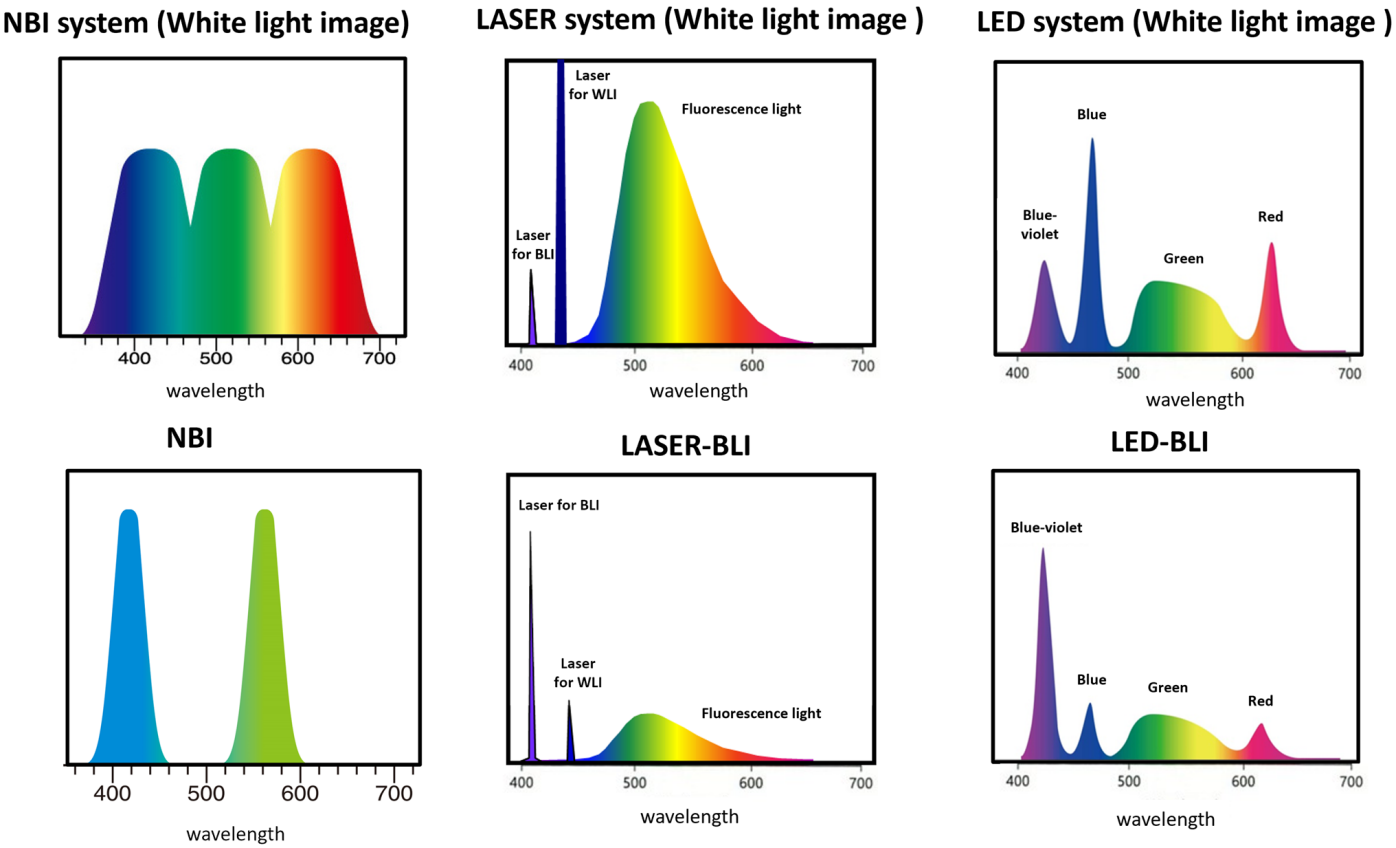
Wavelength of each endoscopy system. NBI figures are provided by Olympus. Laser-BLI and LED-BLI figures are provided by Fujifilm

In the EVIS LUCERA ELITE system (Olympus Medical Systems, Tokyo, Japan), optical filters that allowed narrow band light to pass at wavelengths of 415 and 540 nm were mechanically inserted between a xenon lamp and a RGB rotation filter (Fig. 1).

NBI images were obtained using an PCF-H290ZI endoscope and an EVIS LUCERA ELITE endoscopic system consisting of a CV-290 processor and a CLV-290 light source (Olympus Medical Systems). When using NBI, the structure enhancement function was fixed at level B8.

#### Laser-BLI

The LASEREO endoscopic system (Fujifilm, Tokyo, Japan) was developed as a laser endoscopic system with a light source comprising 2 lasers with wavelengths of 410 and 450 nm. The 450-nm-wavelength laser excites the white light phosphor and produced fluorescent light for standard observations. The 410-nm-wavelength laser was for Laser-BLI. Compared with white light, Laser-BLI improved the contrast of hemoglobin, and obtained selective information on the mucosal superficial vessels and structures (Fig. 1).

Laser-BLI images were obtained using an EC-L600ZP7 endoscope and the LASEREO endoscopic system consisting of a VP-7000 processor and an LL-7000 light source (Fujifilm). When using Laser-BLI, the structure enhancement function was fixed at level B8.

#### LED-BLI

The ELUXEO endoscopic system (Fujifilm) with LEDs of four colors–blue–violet (~410 nm), blue (~450 nm), green (500–600 nm), and red (~630 nm)–was released as a novel endoscopic system in the USA and Europe, where laser endoscopes have not been approved for use. This system enabled BLI with an LED light source instead of a laser light source. By controlling each of the four types of LEDs independently, the technique made it possible to produce light with an appropriate ratio for IEE. LED-BLI was expected to have the same diagnostic performance as Laser-BLI (Fig. 1).

LED-BLI images were obtained using an EC-760ZP endoscope and the ELUXEO endoscopic system consisting of a VP-7000 processor and BL-7000 light source (Fujifilm). When using LED-BLI, the structure enhancement function was fixed at level B8.

### Endoscopic resection

Bowel preparation for the procedure was initiated 1 day prior to ER. Each patient was instructed to consume a low-residue diet and take 5 mg oral sodium picosulfate on the evening before ER. On the day of ER, each patient was given 1500 ml polyethylene glycol (PEG). If the stools were not sufficiently clear, an additional 500 ml PEG was given to ensure sufficient bowel cleaning.

Cecum intubation was verified by identification of the appendiceal orifice and ileocecal valve. The location, size and macroscopic type of all the detected lesions were documented according to the Paris Classification [14]. Tumor resection was performed by polypectomy, EMR or ESD according to tumor size and type. To minimize the endoscopists’ bias, all procedures were performed by endoscopy specialists who had performed more than 500 experiences in each IEE procedure.

### Outcomes

The primary outcome measure was the accuracy of each diagnostic category in NICE and JNET for each endoscopic system. According to previous reports, each endoscopic system has high diagnostic accuracy; Here, we investigated non-inferiority of Laser-BLI and LED-BLI compared with NBI according to NICE and JNET classifications. The non-inferiority margin was set at up to − 10% of the diagnostic accuracy of NBI. Secondary endpoints were sensitivity, specificity, positive predictive value (PPV) and negative predictive value (NPV) in each categorical diagnosis.

### Randomization and masking

The investigators reported the patients’ details to the central registration center via fax. After an eligibility check, the patients were randomly assigned to NBI, Laser-BLI or LED-BLI at the central registration center by a computer program. Patients underwent ER by one of three endoscopy devices. All colorectal tumors were photographed using each IEE (NBI, Laser-BLI or LED-BLI), resected and diagnosed pathologically.

We selected high-resolution JPEG images of colorectal tumors that were taken by each observation mode. Information about the system used (NBI, Laser-BLI or LED-BLI) in the images was deleted in order to reduce bias. We cropped the original images to a square shape and deleted endoscopic mask and other peripheral information. The resolution of cropped images was unified to 415×415 pixels per inch (ppi) for fair evaluation (Supplemental Figure 3).

Cropped images were sent to blinded external evaluators (TS, TM, SO and ST) and diagnosed according to NICE and JNET classification.

### Sample size estimation

In a previous NBI study for colorectal tumor diagnosis, accuracy was ~90% for JNET 1, ~80% for JNET 2A, ~70% for JNET 2B and ~90% for JNET 3 [15]. Assuming that Laser-BLI and LED-BLI are also almost equivalent to NBI, the required number of lesions was calculated with a non-inferiority margin of 10%, an α error of 0.05, and a power of 80% to show non-inferiority to NBI, resulting in 180 lesions in each group. We assumed that some inappropriate images would be excluded, and proposed to collect a total of 200 images in each group. If we were to remove two to three polyps per polypectomy, we would need a total of 200–300 patients, so we decided to collect 240 patients, with 80 patients in each group.

### Statistical analysis

The results are presented as means or medians (± standard deviation or range) for the quantitative data, and as frequencies (percentages) for the categorical data. Categorical data were analyzed using the *χ*^2^ test or Fisher’s exact test, as appropriate. Data showing normal distribution were compared by the t-test, and those showing non-normal distribution were compared by the Mann-Whitney *U* test, to assess the statistical significance of differences. Intra-observer reliability was calculated by Cronbach’s alpha. *p*<0.05 was considered statistically significant. All statistical analyses were carried out using JMP pro, version 15 (SAS Institute, Cary, NC, USA).

All authors had access to the study data and reviewed and approved the final manuscript.

## Results

### Study flow

**Fig. 2:**
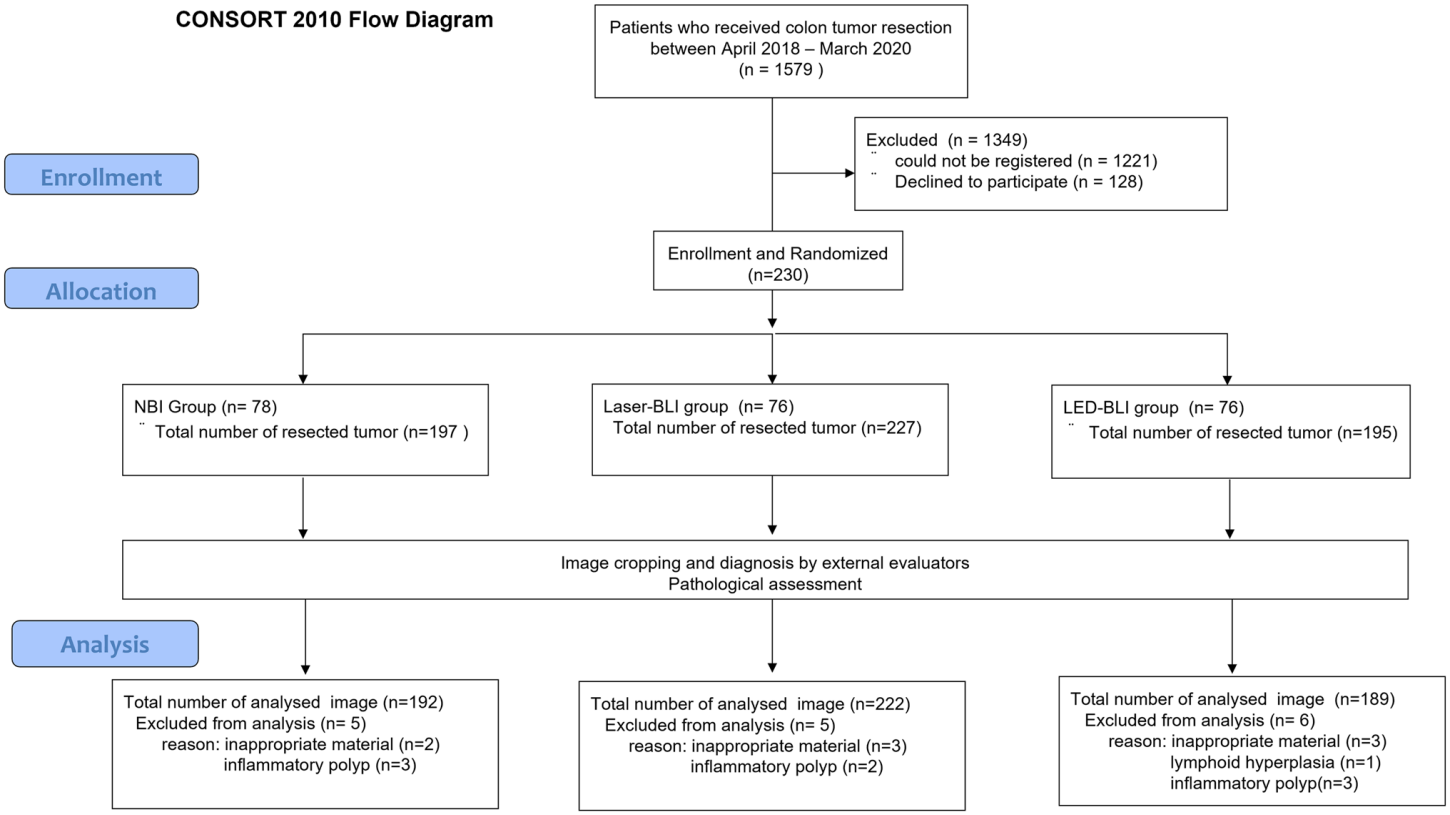
Flow diagram of patients and colorectal tumors included in this study

From April 2018 to March 2020, a total 1579 patients underwent polypectomy. After checking the eligibility and informed consent for the study, 230 patients were enrolled. The reasons for not being included were that 1221 patients could not be registered because of lack of medical resources (endoscopist or endoscope procedure) and 128 patients did not want to participate in the study. The remaining 230 patients were randomized and 78 were assigned to the NBI group, 76 to the Laser-BLI group and 76 to the LED-BLI group. The study participants underwent ER and 619 tumors were totally resected (NBI: 197, LASER-BLI: 227, and LED-BLI: 195) and evaluated by external evaluators. The study flow is shown in Fig. 2.

### Patient and colonic tumor characteristics

The clinical characteristics of the patients are presented in Table 1. There was no significant difference in patient characteristics between the NBI, Laser-BLI and LED-BLI groups. In the NBI group, 192 colonic tumors were resected and sent for external review, along with 222 in the Laser-BLI group, and 189 in LED-BLI group. The colonic tumor characteristics are also presented in Table 1. The composition of the resected tumors was as follow: 146 HP+SSL, 353 LGD, 103 HGD+s-SM cancer and 1 d-SM cancer.

**Table 1:** Patients and resected colonic tumor in each group

	NBI group	Laser-BLI group	LED-BLI group	*P* value
Patient characteristic				
Patient number	78	76	76	
Age (mean ± SD)	70.6±9.4	69.1±11.0	69.7±10.3	0.66
Sex (male : female)	58 : 20	49 : 27	53 : 23	0.41
Resected tumor characteristic				
Total number of resected tumors	192	222	189	
Tumor pathology				0.21
Hyperplastic polyp + SSL	35	62	49	
Low-grade dysplasia	117	126	110	
High-grade dysplasia/s-SM invasion cancer	39	34	30	
d-SM invasive cancer	1	0	0	
Tumor location				0.70
Cecum	23	16	17	
Ascending colon	52	59	46	
Transverse colon	36	33	36	
Descending colon	17	20	13	
Sigmoid colon	38	58	47	
Rectum	26	36	30	
Tumor size (mm) (median, range)	6 (2-45)	6 (2-60)	5 (2-70)	0.30
Tumor morphology				0.17
Is	30	28	17	
Isp	26	38	30	
Ip	11	10	6	
IIa	123	140	135	
IIb	0	4	0	
IIc	2	2	1	

*NBI* narrow band imaging, *Laser-BLI* blue laser imaging, *LED-BLI* blue light imaging, *SSL* sessile serrated lesion, *s-SM* shallow submucosal, *d-SM* deep submucosal

### Comparison of the external evaluation for each procedure

**Fig. 3:**
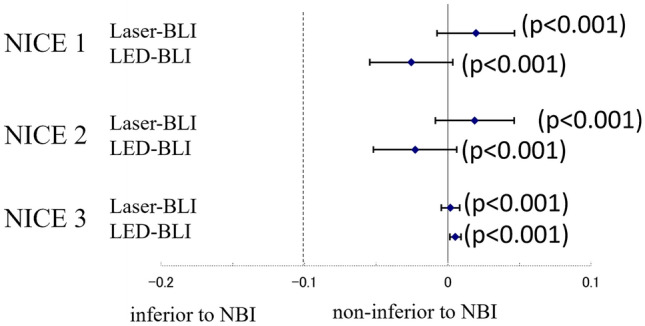
Noninferiority analysis of Laser-BLI and LED-BLI compared with NBI in NICE classification

The cumulative results of NBI for the four evaluators using the NICE classification are shown in Table 2. The diagnostic accuracy for NICE 1, 2 and 3 was 90.6% (95% CI, 88.5%–92.7%), 90.3% (95% CI, 88.2%–92.4%) and 99.5% (95% CI, 99.0%–100.0%), respectively. The sensitivity, specificity, PPV and NPV of each NICE classification using NBI are also shown in Table 2. The cumulative results of Laser-BLI for the four evaluators using the NICE classification are shown in Table 2. The diagnostic accuracy of NICE 1, 2 and 3 was 92.5% (95% CI, 90.8%–94.3%), 92.2% (95% CI, 90.4%–94.0%) and 99.7% (95% CI, 99.3%–100.0%), respectively. The sensitivity, specificity, PPV and NPV of each NICE classification using Laser-BLI are also shown in Table 2. The cumulative results for Laser-BLI for the four evaluators using the NICE classification are shown in Table 2, the accuracy of NICE 1, 2 and 3 was 88.0% (95% CI, 85.7%–90.3%), 88.0% (95% CI, 85.7%–90.3%) and 100% (95% CI, 100%–100%), respectively. Comparison of the accuracy of each diagnostic category in NICE is shown in Fig. 3. Laser-BLI and LED-BLI revealed non-inferiority to NBI for all of NICE 1–3 (*p*<0.001). Intra-observer reliabilities (Cronbach’s alpha) of NBI, Laser-BLI and LED-BLI were 0.961, 0.962 and 0.917, respectively (Table 2). The results of each external evaluators are shown in Supplemental. Table 1

**Table 2:** Relationship between NICE classification and pathological findings, and diagnostic performance of NBI, Laser-BLI and LED-BLI

			Pathological findings			Diagnostic performance					
	NICE classification	N (%)	HP/SSL	LGD+HGD+s-SM	d-SM	Sensitivity	Specificity	PPV	NPV	Accuracy	Cronbach’s alpha
NBI	Type 1	176 (23.0)	125	51	0	85.6 (79.9-91.3)	91.8 (89.6-93.9)	71.0 (64.3-77.7)	96.4 (94.9-97.9)	90.6 (88.5-92.7)	
	Type 2	583 (76.2)	21	562	0	91.4 (89.2-93.6)	88.4(83.2-93.6)	96.4 (94.9-97.9)	70.1(63.5-76.7)	90.3 (88.2-92.4)	
	Type 3	6 (0.8)	0	2	4	100 (100.0-100.0)	99.7 (99.4-100.1)	66.7 (28.9-104.4)	100 (100.0-100.0)	99.5 (99.0-100.0)	
	Total	765 (100)	146	615	4						0.961
Laser- BLI	Type 1	231 (26.5)	210	21	0	82.7 (78.0-87.3)	96.6 (95.2-98.0)	90.9 (87.2-94.6)	93.1 (91.2-95.1)	92.5 (90.8-94.3)	
	Type 2	638 (73.2)	44	594	0	96.1 (94.6-97.6)	82.7 (78.0-87.3)	93.1 (91.1-95.1)	89.7 (85.9-93.6)	92.2 (90.4-94.0)	
	Type 3	3 (0.3)	0	3	0	N/A	99.7 (99.3-100.0)	0 (0.0-0.0)	100 (100.0-100.0)	99.7 (99.3-100.0)	
	Total	872 (100)	254	618	0						0.962
LED- BLI	Type 1	231 (31.0)	179	57	0	84.0 (79.1-89.0)	89.6 (87.0-92.2)	75.8 (70.4-81.3)	93.5 (91.4-95.6)	88.0 (85.7-90.3)	
	Type 2	525 (69.0)	34	491	0	89.6 (87.0-92.2)	84.0 (79.1-89.0)	93.5 (91.4-95.6)	75.8 (70.4-81.3)	88.0 (85.7-90.3)	
	Type 3	0 (0)	0	0	0	N/A	100 (100.0-100.0)	N/A	100 (100.0-100.0)	100 (100.0-100.0)	
	Total	761 (100)	213	548	0						0.917

This table shows cumulative results of the four evaluators

*NBI* narrow band imaging, *Laser-BLI* blue laser imaging, *LED-BLI* blue light imaging, *NICE* NBI international colorectal endoscopic, *SSL* sessile serrated lesion, *s-SM* shallow submucosal, *d-SM* deep submucosal, *PPV* positive predictive value, *NPV* negative predictive value

**Fig. 4:**
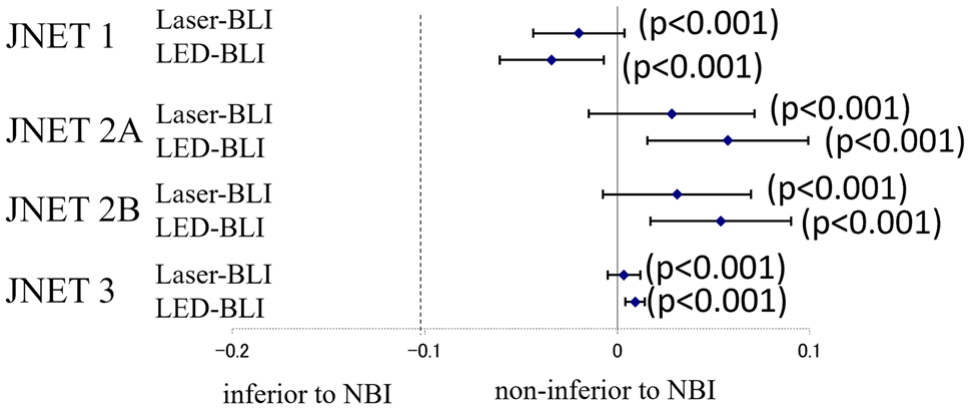
Noninferior analysis of Laser-BLI and LED-BLI compared with NBI in JNET classification

The cumulative results for NBI for the four evaluators using the JNET classification are shown in Table 3. The diagnostic accuracy of JNET 1, 2A, 2B and 3 was 94.6% (95% CI, 93.0%–96.2%), 75.5% (95% CI, 72.5%–78.6%) and 79.2% (95% CI, 76.3%–82.1%) and 99.1% (95% CI, 98.4%–99.8%), respectively. The sensitivity, specificity, PPV and NPV of each JNET classification using NBI are also shown in Table 3. The cumulative results for Laser-BLI for the four evaluators using the JNET classification are shown in Table 3. The diagnostic accuracy of JNET 1, 2A, 2B and 3 was 92.6% (95% CI, 90.9%–94.3%), 74.8% (95% CI, 71.9%–77.7%) and 82.3% (95% CI, 79.8%–84.9%) and 99.4% (95% CI, 98.9%–99.9%), respectively. The sensitivity, specificity, PPV and NPV for each JNET classification using LASER-BLI are also shown in Table 3. The cumulative results for LED-BLI for the four evaluators using the JNET classification are shown in Table 3. The diagnostic accuracy for JNET 1, 2A, 2B and 3 was 91.2% (95% CI, 89.1%–93.3%), 76.1% (95% CI, 73.0%–79.2%) and 84.6% (95% CI, 82.0%–87.2%) and 100% (95% CI, 100%–100%), respectively. The sensitivity, specificity, PPV and NPV of each JNET classification using LASER-BLI are also shown in Table 3. Comparison of the accuracy of each diagnostic category in JNET is shown in Fig. 4. Laser-BLI and LED-BLI revealed non-inferiority to NBI in all JNET 1, 2A, 2B and 3 (*p*<0.001). Intra-observer reliabilities (Cronbach’s alpha) of NBI, Laser-BLI and LED-BLI were 0.920, 0.889 and 0.922, respectively (Table 2). The results for each external evaluators are shown in Supplemental Table 3.

**Table 3.** Relationship between JNET classification and pathological findings, and diagnostic performance of NBI, Laser-BLI and LED-BLI

			Pathological findings				Diagnostic performance (%, 95% CI)					
	JNET classification	N (%)	HP/SSL	LGD	HGD+s-SM	d-SM	Sensitivity	Specificity	PPV	NPV	Accuracy	Cronbach’s alpha
NBI	Type 1	123 (16.2)	111	9	3	0	84.0 (79.1-89.0)	89.6 (87.0-92.2)	90.2 (85.0-95.5)	95.4 (93.8-97.1)	94.6 (93.0-96.2)	
	Type 2A	479 (63.0)	29	379	71	0	81.5 (78.0-85.0)	66.1 (60.7-71.5)	79.1 (75.5-82.8)	69.4 (64.0-74.8)	75.5 (72.5-78.6)	
	Type 2B	151 (19.9)	0	77	72	2	47.7 (39.7-55.6)	87.0 (84.4-89.7)	47.7 (39.7-55.6)	87.0 (84.4-89.7)	79.2 (76.3-82.1)	
	Type 3	7 (0.9)	0	0	5	2	50.0 (10.0-99.0)	99.3 (98.8-99.9)	28.6 (-4.9-62.0)	99.7 (99.4-100.1)	99.1 (98.4-99.8)	
	Total	760 (100)	140	465	151	4						0.920
Laser- BLI	Type 1	217 (25.1)	200	17	0	0	81.0 (76.1-85.9)	97.2 (96.0-98.5)	92.2 (88.6-95.7)	92.7 (90.7-94.7)	92.6 (90.9-94.3)	
	Type 2A	512 (59.2)	47	391	74	0	80.1 (76.6-83.7)	67.9 (63.2-72.6)	79.1 (75.5-82.8)	72.5 (67.9-77.2)	74.8 (71.9-77.7)	
	Type 2B	131 (15.1)	0	77	54	0	41.5 (33.1-50.0)	89.5 (87.3-91.7)	41.2 (32.8-49.7)	89.6 (87.4-91.8)	82.3 (79.8-84.9)	
	Type 3	5 (0.6)	0	3	2	0	N/A	99.4 (98.9-99.9)	0 (0.0-0.0)	100 (100.0-100.0)	99.4 (98.9-99.9)	
	Total	865 (100)	247	488	130	0						0.889
LED-BLI	Type 1	195 (26.8)	159	36	0	0	85.5 (80.4-90.5)	93.3 (91.2-95.4)	81.5 (76.1-87.0)	94.7 (92.8-96.6)	91.2 (89.1-93.3)	
	Type 2A	440 (60.5)	27	346	67	0	81.2(77.5-84.9)	69.0 (63.8-74.2)	78.6 (74.8-82.5)	72.1 (66.9-77.3)	76.1 (73.0-79.2)	
	Type 2B	92 (12.7)	1	44	47	0	41.2 (32.2-50.3)	92.8 (90.8-94.9)	51.1 (40.9-61.3)	89.4 (87.1-91.8)	84.6 (82.0-87.2)	
	Type 3	0 (0)	0	0	0	0	N/A	100 (100.0-100.0)	N/A	100 (100.0-100.0)	100 (100.0-100.0)	
	Total	727 (100)	186	426	114	0						0.926

This table shows cumulative results of the four evaluators

*NBI* narrow band imaging, *Laser-BLI* blue laser imaging, *LED-BLI* blue light imaging, *JNET* Japanese *NBI* expert team, *SSL* sessile serrated lesion, *s-SM* shallow submucosal, *d-SM* deep submucosal, *PPV* positive predictive value, *NPV* negative predictive value

## Discussion

This study is first prospective randomized controlled trial to compare directly the diagnostic accuracy of NBI, Laser-BLI and LED-BLI for colorectal tumor using NICE and JNET classifications. In the analysis of NICE classification, all three IEE devices had high accuracy in all diagnostic categories. In the analysis of JNET classification, all three IEE devices had ~90% or higher accuracy in JNET 1 and JNET 3, and 70–80% accuracy in JNET 2A and JNET 2B. Laser-BLI and LED-BLI showed noninferiority for NBI for NICE and JNET classifications. Furthermore, each IEE showed a high intra-observer reliability rate both in the NICE and JNET classifications. However, there were few d-SM cancer cases, which had insufficient power to confirm the non-inferiority of Laser-BLI and LED-BLI to NBI for NICE 3 and JNET 3.

NBI and Laser-BLI have already been compared for diagnosis of gastric lesions. Kimura et al. reported that Laser-BLI was superior to NBI in delineating shallow glandular ducts and better at diagnosing gastric lesions. It is suggested that this is because NBI changes the spectral features by narrowing the spectral transmittance band using filters tuned to both 415 and 540 nm, while Laser-BLI combines 410 and 450 nm lasers, shorter wavelengths of light, and is therefore superior in delineating shallow glandular ducts [16, 17]. A comparison of Laser-BLI and LED-BLI reported that LED-BLI also made combined 410 and 450 nm LEDs and showed non-inferiority for diagnostic accuracy of early gastric cancer [18]. When we look at the present results from that perspective, the diagnostic accuracy of Laser-BLI and LED-BLI for JNET 2B was bit superior to that of NBI. This may be because both Laser-BLI and LED-BLI can reveal well shallow glandular ducts even in the colon. On the other hand, NBI was bit superior to Laser-BLI and LED-BLI for JNET 1. This may have a similar explanation. Generally, lesions diagnosed to JNET 1, have invisible vessels and regular dark or white spots on their surface, similar to the surrounding normal mucosa. However, as previously described, both Laser-BLI and LED-BLI show shallow glandular ducts and vessels and surface patterns can be over-diagnosed, may explain why Laser-BLI and LED-BLI had less accuracy compared with NBI for JNET 1 lesions.

Recently, a European group proposed the BASIC (BLI adenoma serrated international classification) classification for colorectal polyp characterization with LED-BLI [7]. This new classification that incorporates both morphological and pit/vascular findings shows a high concordance among endoscopists for most of the findings [19, 20]. BASIC is similar to JNET classification, and we revealed that LED-BLI had high diagnostic accuracy using JNET classification in this study. Four evaluators revealed high intra-observer reliability rate in all endoscopic procedures, NBI, Laser-BLI and LED-BLI. Therefore, we suggest that, although NICE and JNET classifications are used for NBI, they could be adapted to both Laser-BLI and LED-BLI.

In recent years, the European Society of Gastrointestinal Endoscopy (ESGE) 2019 guidelines [21] and American Society of Gastrointestinal Endoscopy (ASGE) 2020 guidelines [22] recommended the use of NBI for prediction of histopathological diagnosis and tumor invasion. BLI was described as one of the optical diagnosis options at this point, because it has not been on the market for a long time and there have been few meta- analyses. We believe that future guidelines will establish a recommendation for Laser-BLI and LED-BLI.

There were several limitations to this study. First, ER was conducted in a single center and study participants were patients who underwent ER. Therefore, many of the resected tumors were LGD or HP (NICE1, 2 or JNET 1, 2A), and there were few cases of HGD or d-SM cancer. As a result, the prior probability of HGD and d-SM cancer were low, leading to a high rate of accuracy in NICE 3, JNET 2B and JNET 3. Generally, most patients who undergo ER have LGD. To confirm the noninferiority of Laser-BLI and LED-BLI to NBI in NICE 3 or JNET 2B or 3, further study is required with more cases of HGD, s-SM and d-SM invasive cancer. Tumor distribution in each group was different and this might have affected diagnostic accuracy and PPV. To overcome those limitation, a trial with a group with the same distribution of lesions or a trial that observes the same lesions for each IEE procedure is needed. Second, we cropped original endoscopic images and unified them to 415×415 ppi for fair evaluation. Therefore, image quality was worse than original images and this may have affected diagnostic accuracy. Third, external evaluators judged only one cropped image, which may not have the area of concern, especially large tumor, and this may have affected the results. In clinical practice, endoscopists can diagnose from many types of endoscopic information; therefore, accuracy of actual on-site diagnosis may be better than that of diagnosis from images alone. Third, large number of patients were excluded from this trial because of lack of medical resources, which might have resulted in inclusion bias. Finally, the endoscopists and evaluators in this trial were all experts, and further analysis is needed to establish whether these results can be applied to trainees.

In conclusion, our results indicated that Laser-BLI and LED-BLI had high diagnostic accuracy and noninferiority to NBI, especially for HP/SSL and LGD. Our results are useful to understand the position in the diagnostic accuracy of NBI, Laser-BLI and LED-BLI.

## Supplementary Information

Below is the link to the electronic supplementary material.

Supplementary file1 (PPTX 2862 KB)

## Data Availability

The datasets used and analyzed during the study are available from the corresponding author upon reasonable request.

## Abbreviations

ASGE: American Society of Gastrointestinal Endoscopy
BLI: Blue laser imaging, blue light imaging
CONSORT: Consolidated Standards of Reporting Trials
d-SM: Deep submucosal
EMR: endoscopic mucosal resection
ER: Endoscopic resection
ESD: Endoscopic submucosal dissection
ESGE: European Society of Gastrointestinal Endoscopy
HGD: High-grade dysplasia
HP: Hyperplastic polyp
IEE: Image-enhanced endoscopy
JNET: Japanese NBI expert team
LED: Light emitting diode
LGD: Low-grade dysplasia
NBI: Narrow band imaging
NICE: NBI International Colorectal Endoscopic
NPV: Negative predictive value
PEG: Polyethylene glycol
Ppi: Pixels per inch
PPV: positive predictive value
SSL: Sessile serrated lesion
s-SM: Shallow submucosal
UMIN: University Hospital Medical Information Network
YCU: Yokohama City University
