# Supplementary material for: Comparison of the diagnostic performance of NBI, Laser-BLI and LED-BLI: a randomized controlled noninferiority trial
Source: Surg Endosc. 2022 Apr 11;36(10):7577–87. doi: 10.1007/s00464-022-09197-8 (PMC9485093; doi:10.1007/s00464-022-09197-8)
Supplement: Supplementary file 1 — Supplementary file1 (PPTX 2862 KB) [file 464_2022_9197_MOESM1_ESM.pptx]

## Slide 1
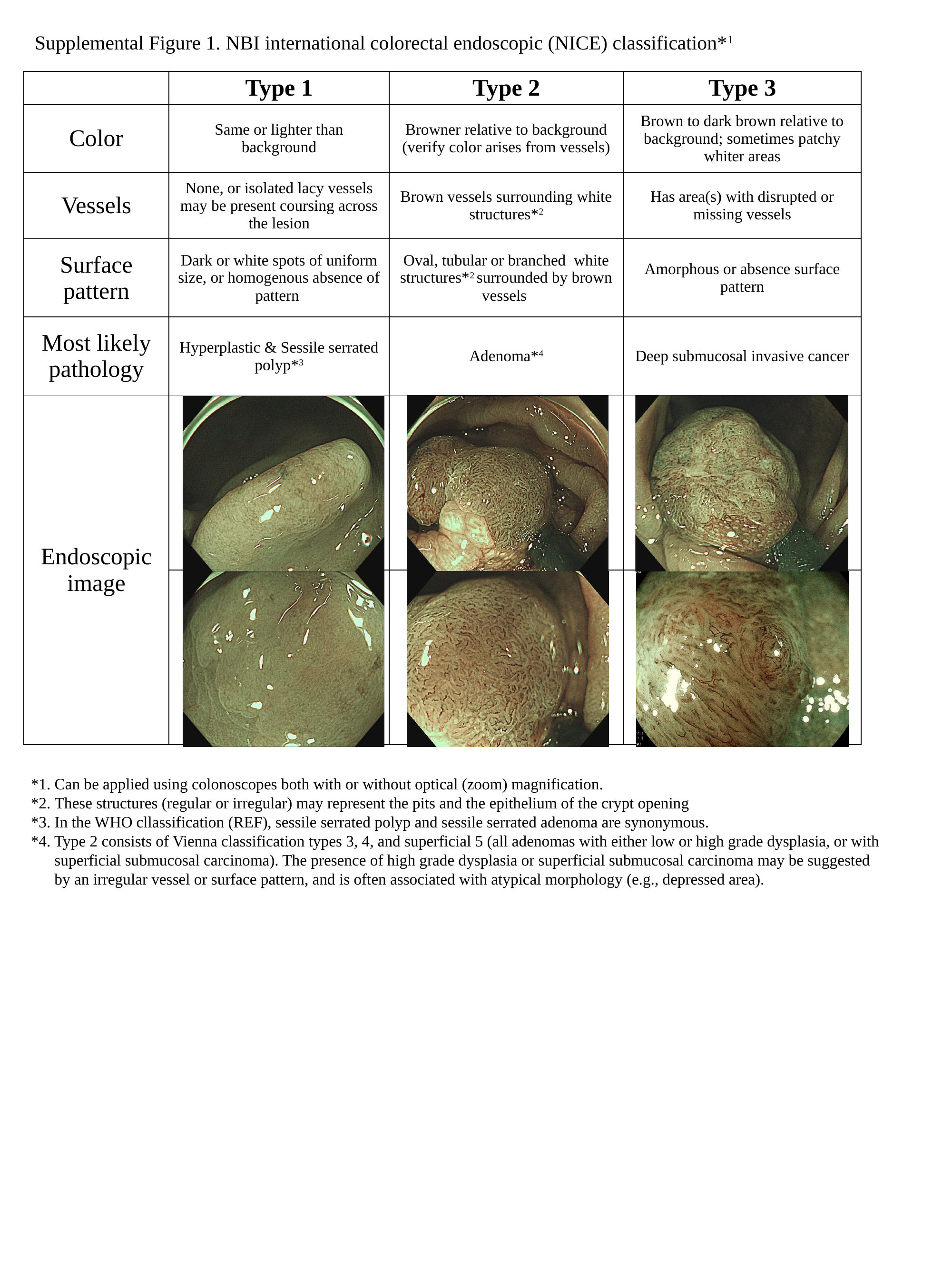

Supplemental Figure 1. NBI international colorectal endoscopic (NICE) classification*1
| | Type 1 | Type 2 | Type 3 |
| --- | --- | --- | --- |
| Color | Same or lighter than background | Browner relative to background (verify color arises from vessels) | Brown to dark brown relative to background; sometimes patchy whiter areas |
| Vessels | None, or isolated lacy vessels may be present coursing across the lesion | Brown vessels surrounding white structures\*2 | Has area(s) with disrupted or missing vessels |
| Surface pattern | Dark or white spots of uniform size, or homogenous absence of pattern | Oval, tubular or branched white structures\*2 surrounded by brown vessels | Amorphous or absence surface pattern |
| Most likely pathology | Hyperplastic & Sessile serrated polyp\*3 | Adenoma\*4 | Deep submucosal invasive cancer |
| Endoscopic image | | | |
| | | | |
*1. Can be applied using colonoscopes both with or without optical (zoom) magnification.
*2. These structures (regular or irregular) may represent the pits and the epithelium of the crypt opening
*3. In the WHO cllassification (REF), sessile serrated polyp and sessile serrated adenoma are synonymous.
*4. Type 2 consists of Vienna classification types 3, 4, and superficial 5 (all adenomas with either low or high grade dysplasia, or with
 superficial submucosal carcinoma). The presence of high grade dysplasia or superficial submucosal carcinoma may be suggested
 by an irregular vessel or surface pattern, and is often associated with atypical morphology (e.g., depressed area).

## Slide 2
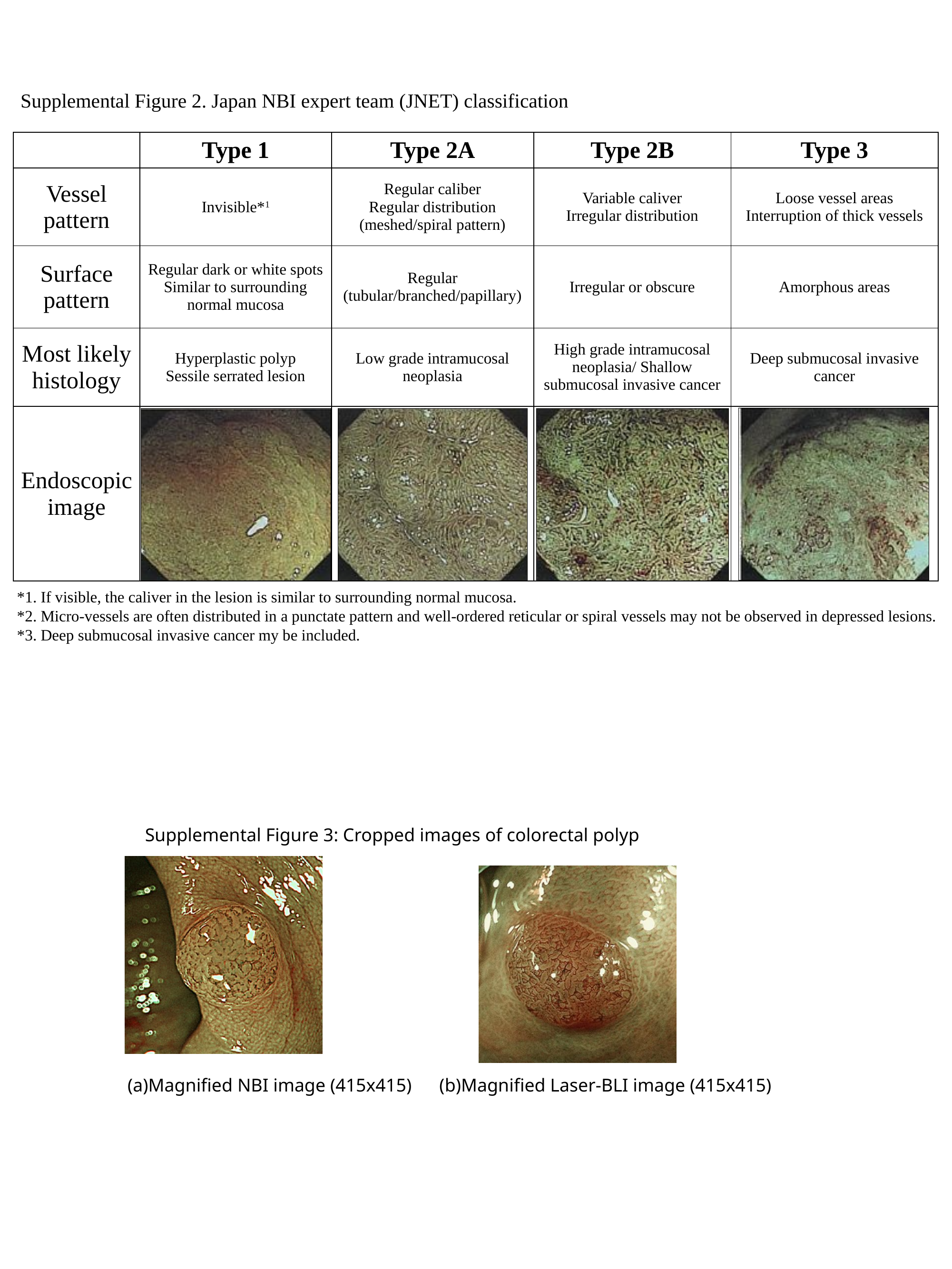

Supplemental Figure 2. Japan NBI expert team (JNET) classification
| | Type 1 | Type 2A | Type 2B | Type 3 |
| --- | --- | --- | --- | --- |
| Vessel pattern | Invisible\*1 | Regular caliber Regular distribution (meshed/spiral pattern) | Variable caliver Irregular distribution | Loose vessel areas Interruption of thick vessels |
| Surface pattern | Regular dark or white spots Similar to surrounding normal mucosa | Regular (tubular/branched/papillary) | Irregular or obscure | Amorphous areas |
| Most likely histology | Hyperplastic polyp Sessile serrated lesion | Low grade intramucosal neoplasia | High grade intramucosal neoplasia/ Shallow submucosal invasive cancer | Deep submucosal invasive cancer |
| Endoscopic image | | | | |
*1. If visible, the caliver in the lesion is similar to surrounding normal mucosa.
*2. Micro-vessels are often distributed in a punctate pattern and well-ordered reticular or spiral vessels may not be observed in depressed lesions.
*3. Deep submucosal invasive cancer my be included.
Supplemental Figure 3: Cropped images of colorectal polyp
(a)Magnified NBI image (415x415) (b)Magnified Laser-BLI image (415x415)

## Slide 3
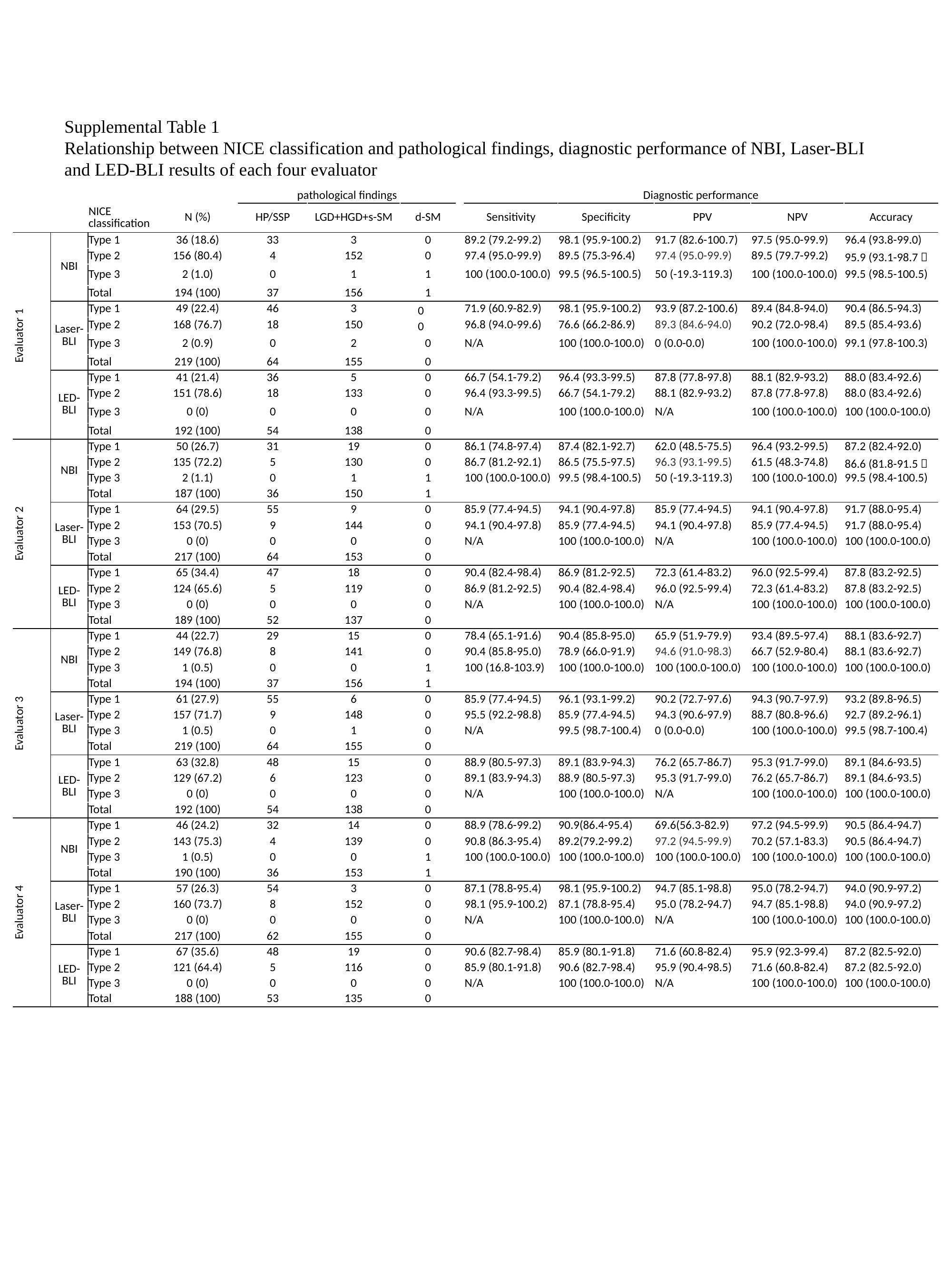

Supplemental Table 1
Relationship between NICE classification and pathological findings, diagnostic performance of NBI, Laser-BLI
and LED-BLI results of each four evaluator
| | | | | pathological findings | | | | Diagnostic performance | | | | |
| --- | --- | --- | --- | --- | --- | --- | --- | --- | --- | --- | --- | --- |
| | | NICE classification | N (%) | HP/SSP | LGD+HGD+s-SM | d-SM | | Sensitivity | Specificity | PPV | NPV | Accuracy |
| Evaluator 1 | NBI | Type 1 | 36 (18.6) | 33 | 3 | 0 | | 89.2 (79.2-99.2) | 98.1 (95.9-100.2) | 91.7 (82.6-100.7) | 97.5 (95.0-99.9) | 96.4 (93.8-99.0) |
| | | Type 2 | 156 (80.4) | 4 | 152 | 0 | | 97.4 (95.0-99.9) | 89.5 (75.3-96.4) | 97.4 (95.0-99.9) | 89.5 (79.7-99.2) | 95.9 (93.1-98.7） |
| | | Type 3 | 2 (1.0) | 0 | 1 | 1 | | 100 (100.0-100.0) | 99.5 (96.5-100.5) | 50 (-19.3-119.3) | 100 (100.0-100.0) | 99.5 (98.5-100.5) |
| | | Total | 194 (100) | 37 | 156 | 1 | | | | | | |
| | Laser- BLI | Type 1 | 49 (22.4) | 46 | 3 | 0 | | 71.9 (60.9-82.9) | 98.1 (95.9-100.2) | 93.9 (87.2-100.6) | 89.4 (84.8-94.0) | 90.4 (86.5-94.3) |
| | | Type 2 | 168 (76.7) | 18 | 150 | 0 | | 96.8 (94.0-99.6) | 76.6 (66.2-86.9) | 89.3 (84.6-94.0) | 90.2 (72.0-98.4) | 89.5 (85.4-93.6) |
| | | Type 3 | 2 (0.9) | 0 | 2 | 0 | | N/A | 100 (100.0-100.0) | 0 (0.0-0.0) | 100 (100.0-100.0) | 99.1 (97.8-100.3) |
| | | Total | 219 (100) | 64 | 155 | 0 | | | | | | |
| | LED- BLI | Type 1 | 41 (21.4) | 36 | 5 | 0 | | 66.7 (54.1-79.2) | 96.4 (93.3-99.5) | 87.8 (77.8-97.8) | 88.1 (82.9-93.2) | 88.0 (83.4-92.6) |
| | | Type 2 | 151 (78.6) | 18 | 133 | 0 | | 96.4 (93.3-99.5) | 66.7 (54.1-79.2) | 88.1 (82.9-93.2) | 87.8 (77.8-97.8) | 88.0 (83.4-92.6) |
| | | Type 3 | 0 (0) | 0 | 0 | 0 | | N/A | 100 (100.0-100.0) | N/A | 100 (100.0-100.0) | 100 (100.0-100.0) |
| | | Total | 192 (100) | 54 | 138 | 0 | | | | | | |
| Evaluator 2 | NBI | Type 1 | 50 (26.7) | 31 | 19 | 0 | | 86.1 (74.8-97.4) | 87.4 (82.1-92.7) | 62.0 (48.5-75.5) | 96.4 (93.2-99.5) | 87.2 (82.4-92.0) |
| | | Type 2 | 135 (72.2) | 5 | 130 | 0 | | 86.7 (81.2-92.1) | 86.5 (75.5-97.5) | 96.3 (93.1-99.5) | 61.5 (48.3-74.8) | 86.6 (81.8-91.5） |
| | | Type 3 | 2 (1.1) | 0 | 1 | 1 | | 100 (100.0-100.0) | 99.5 (98.4-100.5) | 50 (-19.3-119.3) | 100 (100.0-100.0) | 99.5 (98.4-100.5) |
| | | Total | 187 (100) | 36 | 150 | 1 | | | | | | |
| | Laser- BLI | Type 1 | 64 (29.5) | 55 | 9 | 0 | | 85.9 (77.4-94.5) | 94.1 (90.4-97.8) | 85.9 (77.4-94.5) | 94.1 (90.4-97.8) | 91.7 (88.0-95.4) |
| | | Type 2 | 153 (70.5) | 9 | 144 | 0 | | 94.1 (90.4-97.8) | 85.9 (77.4-94.5) | 94.1 (90.4-97.8) | 85.9 (77.4-94.5) | 91.7 (88.0-95.4) |
| | | Type 3 | 0 (0) | 0 | 0 | 0 | | N/A | 100 (100.0-100.0) | N/A | 100 (100.0-100.0) | 100 (100.0-100.0) |
| | | Total | 217 (100) | 64 | 153 | 0 | | | | | | |
| | LED- BLI | Type 1 | 65 (34.4) | 47 | 18 | 0 | | 90.4 (82.4-98.4) | 86.9 (81.2-92.5) | 72.3 (61.4-83.2) | 96.0 (92.5-99.4) | 87.8 (83.2-92.5) |
| | | Type 2 | 124 (65.6) | 5 | 119 | 0 | | 86.9 (81.2-92.5) | 90.4 (82.4-98.4) | 96.0 (92.5-99.4) | 72.3 (61.4-83.2) | 87.8 (83.2-92.5) |
| | | Type 3 | 0 (0) | 0 | 0 | 0 | | N/A | 100 (100.0-100.0) | N/A | 100 (100.0-100.0) | 100 (100.0-100.0) |
| | | Total | 189 (100) | 52 | 137 | 0 | | | | | | |
| Evaluator 3 | NBI | Type 1 | 44 (22.7) | 29 | 15 | 0 | | 78.4 (65.1-91.6) | 90.4 (85.8-95.0) | 65.9 (51.9-79.9) | 93.4 (89.5-97.4) | 88.1 (83.6-92.7) |
| | | Type 2 | 149 (76.8) | 8 | 141 | 0 | | 90.4 (85.8-95.0) | 78.9 (66.0-91.9) | 94.6 (91.0-98.3) | 66.7 (52.9-80.4) | 88.1 (83.6-92.7) |
| | | Type 3 | 1 (0.5) | 0 | 0 | 1 | | 100 (16.8-103.9) | 100 (100.0-100.0) | 100 (100.0-100.0) | 100 (100.0-100.0) | 100 (100.0-100.0) |
| | | Total | 194 (100) | 37 | 156 | 1 | | | | | | |
| | Laser- BLI | Type 1 | 61 (27.9) | 55 | 6 | 0 | | 85.9 (77.4-94.5) | 96.1 (93.1-99.2) | 90.2 (72.7-97.6) | 94.3 (90.7-97.9) | 93.2 (89.8-96.5) |
| | | Type 2 | 157 (71.7) | 9 | 148 | 0 | | 95.5 (92.2-98.8) | 85.9 (77.4-94.5) | 94.3 (90.6-97.9) | 88.7 (80.8-96.6) | 92.7 (89.2-96.1) |
| | | Type 3 | 1 (0.5) | 0 | 1 | 0 | | N/A | 99.5 (98.7-100.4) | 0 (0.0-0.0) | 100 (100.0-100.0) | 99.5 (98.7-100.4) |
| | | Total | 219 (100) | 64 | 155 | 0 | | | | | | |
| | LED- BLI | Type 1 | 63 (32.8) | 48 | 15 | 0 | | 88.9 (80.5-97.3) | 89.1 (83.9-94.3) | 76.2 (65.7-86.7) | 95.3 (91.7-99.0) | 89.1 (84.6-93.5) |
| | | Type 2 | 129 (67.2) | 6 | 123 | 0 | | 89.1 (83.9-94.3) | 88.9 (80.5-97.3) | 95.3 (91.7-99.0) | 76.2 (65.7-86.7) | 89.1 (84.6-93.5) |
| | | Type 3 | 0 (0) | 0 | 0 | 0 | | N/A | 100 (100.0-100.0) | N/A | 100 (100.0-100.0) | 100 (100.0-100.0) |
| | | Total | 192 (100) | 54 | 138 | 0 | | | | | | |
| Evaluator 4 | NBI | Type 1 | 46 (24.2) | 32 | 14 | 0 | | 88.9 (78.6-99.2) | 90.9(86.4-95.4) | 69.6(56.3-82.9) | 97.2 (94.5-99.9) | 90.5 (86.4-94.7) |
| | | Type 2 | 143 (75.3) | 4 | 139 | 0 | | 90.8 (86.3-95.4) | 89.2(79.2-99.2) | 97.2 (94.5-99.9) | 70.2 (57.1-83.3) | 90.5 (86.4-94.7) |
| | | Type 3 | 1 (0.5) | 0 | 0 | 1 | | 100 (100.0-100.0) | 100 (100.0-100.0) | 100 (100.0-100.0) | 100 (100.0-100.0) | 100 (100.0-100.0) |
| | | Total | 190 (100) | 36 | 153 | 1 | | | | | | |
| | Laser- BLI | Type 1 | 57 (26.3) | 54 | 3 | 0 | | 87.1 (78.8-95.4) | 98.1 (95.9-100.2) | 94.7 (85.1-98.8) | 95.0 (78.2-94.7) | 94.0 (90.9-97.2) |
| | | Type 2 | 160 (73.7) | 8 | 152 | 0 | | 98.1 (95.9-100.2) | 87.1 (78.8-95.4) | 95.0 (78.2-94.7) | 94.7 (85.1-98.8) | 94.0 (90.9-97.2) |
| | | Type 3 | 0 (0) | 0 | 0 | 0 | | N/A | 100 (100.0-100.0) | N/A | 100 (100.0-100.0) | 100 (100.0-100.0) |
| | | Total | 217 (100) | 62 | 155 | 0 | | | | | | |
| | LED- BLI | Type 1 | 67 (35.6) | 48 | 19 | 0 | | 90.6 (82.7-98.4) | 85.9 (80.1-91.8) | 71.6 (60.8-82.4) | 95.9 (92.3-99.4) | 87.2 (82.5-92.0) |
| | | Type 2 | 121 (64.4) | 5 | 116 | 0 | | 85.9 (80.1-91.8) | 90.6 (82.7-98.4) | 95.9 (90.4-98.5) | 71.6 (60.8-82.4) | 87.2 (82.5-92.0) |
| | | Type 3 | 0 (0) | 0 | 0 | 0 | | N/A | 100 (100.0-100.0) | N/A | 100 (100.0-100.0) | 100 (100.0-100.0) |
| | | Total | 188 (100) | 53 | 135 | 0 | | | | | | |

## Slide 4
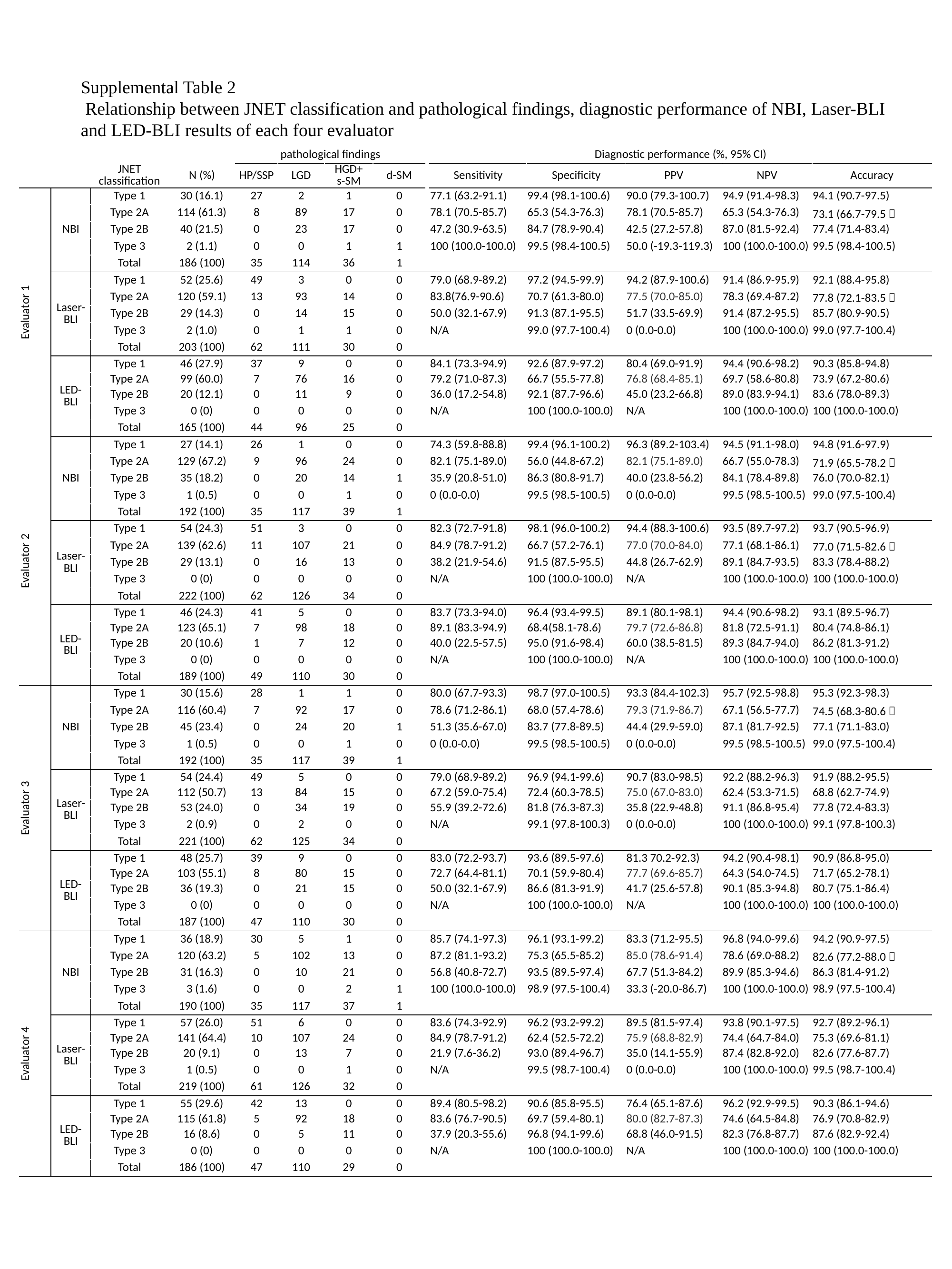

Supplemental Table 2
 Relationship between JNET classification and pathological findings, diagnostic performance of NBI, Laser-BLI
and LED-BLI results of each four evaluator
| | | | | pathological findings | | | | | Diagnostic performance (%, 95% CI) | | | | |
| --- | --- | --- | --- | --- | --- | --- | --- | --- | --- | --- | --- | --- | --- |
| | | JNET classification | N (%) | HP/SSP | LGD | HGD+ s-SM | d-SM | | Sensitivity | Specificity | PPV | NPV | Accuracy |
| Evaluator 1 | NBI | Type 1 | 30 (16.1) | 27 | 2 | 1 | 0 | | 77.1 (63.2-91.1) | 99.4 (98.1-100.6) | 90.0 (79.3-100.7) | 94.9 (91.4-98.3) | 94.1 (90.7-97.5) |
| | | Type 2A | 114 (61.3) | 8 | 89 | 17 | 0 | | 78.1 (70.5-85.7) | 65.3 (54.3-76.3) | 78.1 (70.5-85.7) | 65.3 (54.3-76.3) | 73.1 (66.7-79.5） |
| | | Type 2B | 40 (21.5) | 0 | 23 | 17 | 0 | | 47.2 (30.9-63.5) | 84.7 (78.9-90.4) | 42.5 (27.2-57.8) | 87.0 (81.5-92.4) | 77.4 (71.4-83.4) |
| | | Type 3 | 2 (1.1) | 0 | 0 | 1 | 1 | | 100 (100.0-100.0) | 99.5 (98.4-100.5) | 50.0 (-19.3-119.3) | 100 (100.0-100.0) | 99.5 (98.4-100.5) |
| | | Total | 186 (100) | 35 | 114 | 36 | 1 | | | | | | |
| | Laser- BLI | Type 1 | 52 (25.6) | 49 | 3 | 0 | 0 | | 79.0 (68.9-89.2) | 97.2 (94.5-99.9) | 94.2 (87.9-100.6) | 91.4 (86.9-95.9) | 92.1 (88.4-95.8) |
| | | Type 2A | 120 (59.1) | 13 | 93 | 14 | 0 | | 83.8(76.9-90.6) | 70.7 (61.3-80.0) | 77.5 (70.0-85.0) | 78.3 (69.4-87.2) | 77.8 (72.1-83.5） |
| | | Type 2B | 29 (14.3) | 0 | 14 | 15 | 0 | | 50.0 (32.1-67.9) | 91.3 (87.1-95.5) | 51.7 (33.5-69.9) | 91.4 (87.2-95.5) | 85.7 (80.9-90.5) |
| | | Type 3 | 2 (1.0) | 0 | 1 | 1 | 0 | | N/A | 99.0 (97.7-100.4) | 0 (0.0-0.0) | 100 (100.0-100.0) | 99.0 (97.7-100.4) |
| | | Total | 203 (100) | 62 | 111 | 30 | 0 | | | | | | |
| | LED- BLI | Type 1 | 46 (27.9) | 37 | 9 | 0 | 0 | | 84.1 (73.3-94.9) | 92.6 (87.9-97.2) | 80.4 (69.0-91.9) | 94.4 (90.6-98.2) | 90.3 (85.8-94.8) |
| | | Type 2A | 99 (60.0) | 7 | 76 | 16 | 0 | | 79.2 (71.0-87.3) | 66.7 (55.5-77.8) | 76.8 (68.4-85.1) | 69.7 (58.6-80.8) | 73.9 (67.2-80.6) |
| | | Type 2B | 20 (12.1) | 0 | 11 | 9 | 0 | | 36.0 (17.2-54.8) | 92.1 (87.7-96.6) | 45.0 (23.2-66.8) | 89.0 (83.9-94.1) | 83.6 (78.0-89.3) |
| | | Type 3 | 0 (0) | 0 | 0 | 0 | 0 | | N/A | 100 (100.0-100.0) | N/A | 100 (100.0-100.0) | 100 (100.0-100.0) |
| | | Total | 165 (100) | 44 | 96 | 25 | 0 | | | | | | |
| Evaluator 2 | NBI | Type 1 | 27 (14.1) | 26 | 1 | 0 | 0 | | 74.3 (59.8-88.8) | 99.4 (96.1-100.2) | 96.3 (89.2-103.4) | 94.5 (91.1-98.0) | 94.8 (91.6-97.9) |
| | | Type 2A | 129 (67.2) | 9 | 96 | 24 | 0 | | 82.1 (75.1-89.0) | 56.0 (44.8-67.2) | 82.1 (75.1-89.0) | 66.7 (55.0-78.3) | 71.9 (65.5-78.2） |
| | | Type 2B | 35 (18.2) | 0 | 20 | 14 | 1 | | 35.9 (20.8-51.0) | 86.3 (80.8-91.7) | 40.0 (23.8-56.2) | 84.1 (78.4-89.8) | 76.0 (70.0-82.1) |
| | | Type 3 | 1 (0.5) | 0 | 0 | 1 | 0 | | 0 (0.0-0.0) | 99.5 (98.5-100.5) | 0 (0.0-0.0) | 99.5 (98.5-100.5) | 99.0 (97.5-100.4) |
| | | Total | 192 (100) | 35 | 117 | 39 | 1 | | | | | | |
| | Laser- BLI | Type 1 | 54 (24.3) | 51 | 3 | 0 | 0 | | 82.3 (72.7-91.8) | 98.1 (96.0-100.2) | 94.4 (88.3-100.6) | 93.5 (89.7-97.2) | 93.7 (90.5-96.9) |
| | | Type 2A | 139 (62.6) | 11 | 107 | 21 | 0 | | 84.9 (78.7-91.2) | 66.7 (57.2-76.1) | 77.0 (70.0-84.0) | 77.1 (68.1-86.1) | 77.0 (71.5-82.6） |
| | | Type 2B | 29 (13.1) | 0 | 16 | 13 | 0 | | 38.2 (21.9-54.6) | 91.5 (87.5-95.5) | 44.8 (26.7-62.9) | 89.1 (84.7-93.5) | 83.3 (78.4-88.2) |
| | | Type 3 | 0 (0) | 0 | 0 | 0 | 0 | | N/A | 100 (100.0-100.0) | N/A | 100 (100.0-100.0) | 100 (100.0-100.0) |
| | | Total | 222 (100) | 62 | 126 | 34 | 0 | | | | | | |
| | LED- BLI | Type 1 | 46 (24.3) | 41 | 5 | 0 | 0 | | 83.7 (73.3-94.0) | 96.4 (93.4-99.5) | 89.1 (80.1-98.1) | 94.4 (90.6-98.2) | 93.1 (89.5-96.7) |
| | | Type 2A | 123 (65.1) | 7 | 98 | 18 | 0 | | 89.1 (83.3-94.9) | 68.4(58.1-78.6) | 79.7 (72.6-86.8) | 81.8 (72.5-91.1) | 80.4 (74.8-86.1) |
| | | Type 2B | 20 (10.6) | 1 | 7 | 12 | 0 | | 40.0 (22.5-57.5) | 95.0 (91.6-98.4) | 60.0 (38.5-81.5) | 89.3 (84.7-94.0) | 86.2 (81.3-91.2) |
| | | Type 3 | 0 (0) | 0 | 0 | 0 | 0 | | N/A | 100 (100.0-100.0) | N/A | 100 (100.0-100.0) | 100 (100.0-100.0) |
| | | Total | 189 (100) | 49 | 110 | 30 | 0 | | | | | | |
| Evaluator 3 | NBI | Type 1 | 30 (15.6) | 28 | 1 | 1 | 0 | | 80.0 (67.7-93.3) | 98.7 (97.0-100.5) | 93.3 (84.4-102.3) | 95.7 (92.5-98.8) | 95.3 (92.3-98.3) |
| | | Type 2A | 116 (60.4) | 7 | 92 | 17 | 0 | | 78.6 (71.2-86.1) | 68.0 (57.4-78.6) | 79.3 (71.9-86.7) | 67.1 (56.5-77.7) | 74.5 (68.3-80.6） |
| | | Type 2B | 45 (23.4) | 0 | 24 | 20 | 1 | | 51.3 (35.6-67.0) | 83.7 (77.8-89.5) | 44.4 (29.9-59.0) | 87.1 (81.7-92.5) | 77.1 (71.1-83.0) |
| | | Type 3 | 1 (0.5) | 0 | 0 | 1 | 0 | | 0 (0.0-0.0) | 99.5 (98.5-100.5) | 0 (0.0-0.0) | 99.5 (98.5-100.5) | 99.0 (97.5-100.4) |
| | | Total | 192 (100) | 35 | 117 | 39 | 1 | | | | | | |
| | Laser- BLI | Type 1 | 54 (24.4) | 49 | 5 | 0 | 0 | | 79.0 (68.9-89.2) | 96.9 (94.1-99.6) | 90.7 (83.0-98.5) | 92.2 (88.2-96.3) | 91.9 (88.2-95.5) |
| | | Type 2A | 112 (50.7) | 13 | 84 | 15 | 0 | | 67.2 (59.0-75.4) | 72.4 (60.3-78.5) | 75.0 (67.0-83.0) | 62.4 (53.3-71.5) | 68.8 (62.7-74.9) |
| | | Type 2B | 53 (24.0) | 0 | 34 | 19 | 0 | | 55.9 (39.2-72.6) | 81.8 (76.3-87.3) | 35.8 (22.9-48.8) | 91.1 (86.8-95.4) | 77.8 (72.4-83.3) |
| | | Type 3 | 2 (0.9) | 0 | 2 | 0 | 0 | | N/A | 99.1 (97.8-100.3) | 0 (0.0-0.0) | 100 (100.0-100.0) | 99.1 (97.8-100.3) |
| | | Total | 221 (100) | 62 | 125 | 34 | 0 | | | | | | |
| | LED- BLI | Type 1 | 48 (25.7) | 39 | 9 | 0 | 0 | | 83.0 (72.2-93.7) | 93.6 (89.5-97.6) | 81.3 70.2-92.3) | 94.2 (90.4-98.1) | 90.9 (86.8-95.0) |
| | | Type 2A | 103 (55.1) | 8 | 80 | 15 | 0 | | 72.7 (64.4-81.1) | 70.1 (59.9-80.4) | 77.7 (69.6-85.7) | 64.3 (54.0-74.5) | 71.7 (65.2-78.1) |
| | | Type 2B | 36 (19.3) | 0 | 21 | 15 | 0 | | 50.0 (32.1-67.9) | 86.6 (81.3-91.9) | 41.7 (25.6-57.8) | 90.1 (85.3-94.8) | 80.7 (75.1-86.4) |
| | | Type 3 | 0 (0) | 0 | 0 | 0 | 0 | | N/A | 100 (100.0-100.0) | N/A | 100 (100.0-100.0) | 100 (100.0-100.0) |
| | | Total | 187 (100) | 47 | 110 | 30 | 0 | | | | | | |
| Evaluator 4 | NBI | Type 1 | 36 (18.9) | 30 | 5 | 1 | 0 | | 85.7 (74.1-97.3) | 96.1 (93.1-99.2) | 83.3 (71.2-95.5) | 96.8 (94.0-99.6) | 94.2 (90.9-97.5) |
| | | Type 2A | 120 (63.2) | 5 | 102 | 13 | 0 | | 87.2 (81.1-93.2) | 75.3 (65.5-85.2) | 85.0 (78.6-91.4) | 78.6 (69.0-88.2) | 82.6 (77.2-88.0） |
| | | Type 2B | 31 (16.3) | 0 | 10 | 21 | 0 | | 56.8 (40.8-72.7) | 93.5 (89.5-97.4) | 67.7 (51.3-84.2) | 89.9 (85.3-94.6) | 86.3 (81.4-91.2) |
| | | Type 3 | 3 (1.6) | 0 | 0 | 2 | 1 | | 100 (100.0-100.0) | 98.9 (97.5-100.4) | 33.3 (-20.0-86.7) | 100 (100.0-100.0) | 98.9 (97.5-100.4) |
| | | Total | 190 (100) | 35 | 117 | 37 | 1 | | | | | | |
| | Laser- BLI | Type 1 | 57 (26.0) | 51 | 6 | 0 | 0 | | 83.6 (74.3-92.9) | 96.2 (93.2-99.2) | 89.5 (81.5-97.4) | 93.8 (90.1-97.5) | 92.7 (89.2-96.1) |
| | | Type 2A | 141 (64.4) | 10 | 107 | 24 | 0 | | 84.9 (78.7-91.2) | 62.4 (52.5-72.2) | 75.9 (68.8-82.9) | 74.4 (64.7-84.0) | 75.3 (69.6-81.1) |
| | | Type 2B | 20 (9.1) | 0 | 13 | 7 | 0 | | 21.9 (7.6-36.2) | 93.0 (89.4-96.7) | 35.0 (14.1-55.9) | 87.4 (82.8-92.0) | 82.6 (77.6-87.7) |
| | | Type 3 | 1 (0.5) | 0 | 0 | 1 | 0 | | N/A | 99.5 (98.7-100.4) | 0 (0.0-0.0) | 100 (100.0-100.0) | 99.5 (98.7-100.4) |
| | | Total | 219 (100) | 61 | 126 | 32 | 0 | | | | | | |
| | LED- BLI | Type 1 | 55 (29.6) | 42 | 13 | 0 | 0 | | 89.4 (80.5-98.2) | 90.6 (85.8-95.5) | 76.4 (65.1-87.6) | 96.2 (92.9-99.5) | 90.3 (86.1-94.6) |
| | | Type 2A | 115 (61.8) | 5 | 92 | 18 | 0 | | 83.6 (76.7-90.5) | 69.7 (59.4-80.1) | 80.0 (82.7-87.3) | 74.6 (64.5-84.8) | 76.9 (70.8-82.9) |
| | | Type 2B | 16 (8.6) | 0 | 5 | 11 | 0 | | 37.9 (20.3-55.6) | 96.8 (94.1-99.6) | 68.8 (46.0-91.5) | 82.3 (76.8-87.7) | 87.6 (82.9-92.4) |
| | | Type 3 | 0 (0) | 0 | 0 | 0 | 0 | | N/A | 100 (100.0-100.0) | N/A | 100 (100.0-100.0) | 100 (100.0-100.0) |
| | | Total | 186 (100) | 47 | 110 | 29 | 0 | | | | | | |
